# Causal Relationships Between Blood Metabolites, Cognitive Outcomes, Brain Imaging, and CSF Biomarkers in Alzheimer's Disease: Insights from Digital‐Twins

**DOI:** 10.1002/alz70856_107374

**Published:** 2026-01-09

**Authors:** Apoorva Bharthur Sanjay, Deepanshi Shokeen, Jeanne Latourelle, So‐Youn Shin

**Affiliations:** ^1^ Aitia, Somerville, MA, USA

## Abstract

**Background:**

Alzheimer's Disease (AD) is marked by cognitive decline and metabolic dysregulation, with blood metabolites playing a crucial role in disease progression. However, it remains unclear whether these metabolites actively drive the disease or merely reflect its progression. Identifying causal metabolites and understanding their relationships with biomarkers, cognition, and brain structure could provide new insights into AD pathogenesis and potential therapeutic strategies.

**Method:**

Aitia's AD Digital‐Twin is a virtual model of AD patients and controls, built using ∼59k clinical and multi‐omic variables from ADNI, including 122 serum metabolites profiled by Biocrates AbsoluteIDQ p180 and 20 bile acids from Bile Acids Kit. Using the causal Bayesian network structure within AD Digital‐Twins, we investigated cause‐and‐effect relationships between metabolites, cerebrospinal fluid (CSF) biomarkers, MRI‐derived subcortical measures, and cognitive outcomes.

**Result:**

We identified 44 metabolites (31% of 142) causally related to cognitive outcomes, with nine metabolites driving baseline cognitive function rather than the rate of cognitive decline. Two amino acids, Isoleucine and Lysine, influence both the MMSE score and the thickness of the entorhinal cortex and middle temporal region volume. These amino acids are novel causal drivers of brain structural changes in AD.

Additionally, medium‐chain triglycerides (MCTs) and a sphingomyelin were found to drive changes in middle temporal volume and baseline values, respectively. MCTs are known to enhance brain energy metabolism, and sphingomyelins maintain neuronal membrane integrity, suggesting their central role in neurodegeneration.

Notably, 15 of the 44 cognition‐related metabolites were driven by CSF Tau & pTau, hippocampal volume, and FDG hypometabolism, highlighting that many metabolic changes observed in association studies may be reactions to biomarker changes. The causal relationship with Tau suggests that AD‐related metabolites are primarily influenced by tau pathology rather than amyloid‐beta.

**Conclusion:**

These findings highlight the critical role of Tau pathology, hippocampal atrophy, and glucose hypometabolism in driving metabolic alterations in AD. Some metabolites are causal drivers of cognitive decline and neurodegeneration, while others reflect disease progression, offering potential biomarkers for early diagnosis and treatment. Future work integrating transcriptomic and genotypic data with metabolomics could identify key pathways, advancing our understanding of the genetic and molecular drivers of AD.

